# The Effect of Expertise on Eye Movement Behaviour in Medical Image Perception

**DOI:** 10.1371/journal.pone.0066169

**Published:** 2013-06-13

**Authors:** Raymond Bertram, Laura Helle, Johanna K. Kaakinen, Erkki Svedström

**Affiliations:** 1 Centre for Learning Research and Department of Teacher Education, University of Turku, Turku, Finland; 2 Department of Psychology, University of Turku, Turku, Finland; 3 Department of Diagnostic Radiology, University of Turku, Turku, Finland; University of Leicester, United Kingdom

## Abstract

The present eye-movement study assessed the effect of expertise on eye-movement behaviour during image perception in the medical domain. To this end, radiologists, computed-tomography radiographers and psychology students were exposed to nine volumes of multi-slice, stack-view, axial computed-tomography images from the upper to the lower part of the abdomen with or without abnormality. The images were presented in succession at low, medium or high speed, while the participants had to detect enlarged lymph nodes or other visually more salient abnormalities. The radiologists outperformed both other groups in the detection of enlarged lymph nodes and their eye-movement behaviour also differed from the other groups. Their general strategy was to use saccades of shorter amplitude than the two other participant groups. In the presence of enlarged lymph nodes, they increased the number of fixations on the relevant areas and reverted to even shorter saccades. In volumes containing enlarged lymph nodes, radiologists’ fixation durations were longer in comparison to their fixation durations in volumes without enlarged lymph nodes. More salient abnormalities were detected equally well by radiologists and radiographers, with both groups outperforming psychology students. However, to accomplish this, radiologists actually needed fewer fixations on the relevant areas than the radiographers. On the basis of these results, we argue that expert behaviour is manifested in distinct eye-movement patterns of proactivity, reactivity and suppression, depending on the nature of the task and the presence of abnormalities at any given moment.

## Introduction

In any domain in life, some individuals are better than others are at competing within that domain. People who excel in a certain domain can be termed ‘domain experts’. Expert performance is afforded by extensive domain-related procedural and conceptual knowledge allowing for expert decisions [1]. In most domains, there is a strong visual component to the tasks that need to be performed. For instance, in chess, traffic, biology, sports and medicine, relevant information needs to be extracted from the visual scene, preferably as fast as possible. One may assume that an expert is significantly better in this than a non-expert. From this basic assumption emerges the interesting question as to how the eye-movement behaviour of an expert in a domain-specific scene differs from that of a non-expert.

In general, one may wonder how people manage to quickly extract relevant information from visual scenes in a familiar domain. Wolfe and colleagues [2], [3] suggested that the key to this ability is the use of two, rather than one visual pathway in the visual-search process. They proposed that humans make use of a non-selective visual pathway for extracting global information from across the visual field and a selective visual pathway serving object identification. The latter pathway typically requires foveal inspection. Both pathways are thought to run in parallel, and the non-selective pathway may detect potentially relevant objects and pass them on to the selective pathway for full identification. In this model, the analysis of the entire visual scene is not restricted to the initial stages, but continues during the later stages of analysis.

Several models that are more specifically related to the role of expertise in a certain domain incorporate similar ideas. In the medical domain, the global-focal search model [4], the holistic model of image perception [5] and the two-stage detection model [6] suggest that experts initially perform a quick global analysis of the entire visual scene to determine deviations from the schema of normal anatomic structures. Subsequently, they would saccade towards the location where a mismatch between the schema and the visual scene had been detected to perform a foveal inspection. All these models resonate with the concept of ‘chunking’ introduced by Miller [7] and the theory of long-term working memory introduced by Ericsson and Kintsch [8], as they all assume that experts can quickly retrieve visual information by virtue of the existence of larger constellations of features or larger retrieval structures.

Reingold and Sheridan summarized a large number of eye-movement studies on the role of expertise in visual-scene perception in medicine and chess [9]. In general, these studies show that experts have superior perceptual ability in their own domain. For instance, expert chess players were able to extract useful information from an area of 25 squares around a fixation in real chess-configuration situations, whereas novice and intermediate players could only extract information from about 10 squares [10]. Similarly, several studies imply that medical experts make use of a large visual field in the initial stages of image analysis. For instance, expert radiologists and expert mammographers performed well above chance level in identifying abnormalities under flash viewing conditions in which images were shown for only 200 milliseconds [11], [12]. Given that the brief exposure conditions prevented eye movements towards the abnormalities, it showed that the experts could identify abnormalities without foveal inspection. Moreover, under brief exposure conditions, expert radiologists could detect nodules 15° away from the fixation point [13] and mammographers typically hit upon cancers more than 20° away from the initial fixation point within one second [5]. Other studies show that, in comparison to non-experts, experts typically perform domain-related tasks with fewer fixations [14], [15], longer saccades [14], [16] and less coverage of the image [15], [17]. All these findings can be explained by a larger perceptual span for experts than for non-experts, allowing them to more profoundly make use of the parafoveal/peripheral area in their domain of expertise.

In the current study, we compared the eye-movement behaviour of radiologists, computed-tomography (CT) radiographers and psychology students while they were exposed to CT scans of the abdomen, and performed a visually relatively easy and difficult task [18]. Typically, differences between experts and non-experts increase in more demanding visual settings [19]. The easy task was the detection of visually salient visceral abnormalities; the difficult task was the detection of enlarged lymph nodes (ELNs). ELNs are nodes larger than 1 cm in diameter, calling for further investigation if a definite cause cannot be identified. Nodes of this size can be qualified as prominent, even though there is agreement that nodes of up to 2 cm in diameter may be normally palpable [20]. Determining whether a lymph node is enlarged or not is visually demanding, as the distinction between normal nodes and ELNs is a matter of degree and lymph nodes typically appear in locations with a lot of background noise (e.g., vessels).

In our experiment, we presented multi-slice, axial stack-view images from the upper to the lower part of the abdomen at different framerates, thereby creating dynamic visual scenes. It should be noted though, that the images themselves are cross-sectional images without dynamic elements. Dynamic scenes in the current study are dynamic because an image is replaced by a subsequent one several times a second, leaving a video-like impression.

Obviously, the human perceptual system is more challenged when exposed to dynamic rather than to static scenes. In dynamic scenes, information may only be visible for a short time period and an object of interest may be missed altogether if it is not attended to quickly enough. This implies that dynamic scenes require more goal-directed behaviour. That is, it becomes more crucial to allocate attention to areas in the scene where relevant information may be expected to be. Most of the eye-movement studies on the role of expertise in visual searches have used static scenes (as have the ones discussed above), but there are also a number of studies on dynamic scenes. These studies will be discussed in the next section.

### Eye-movement Research on the Role of Expertise in Dynamic Settings

There are a number of eye-movement studies on dynamic stimuli in non-medical domains. In the biological domain, Jarodzka and colleagues investigated the role of expertise for the description of locomotion patterns in fish [21]. They found that experts attended more to aspects that were relevant for the description of locomotion patterns than did novices, partly making use of knowledge-based shortcuts [22]. Perhaps surprisingly, experts exhibited a more heterogeneous task approach than novices did. Jarodzka et al. noted that this may be have been due to the diversity of their experts, thus making use of more individualized case-based knowledge, whereas novices may have been guided by generic principles such as, for example, visual saliency (for a survey on the role of saliency in visual search, see [23], [24]).

In the domain of traffic, Underwood and colleagues found that the level of expertise interacted with task difficulty in real or simulated car-driving situations [25], [26]. Experts had longer fixation durations than novices when exposed to uneventful rural roads, but they made significantly more and shorter-lasting fixations when driving in a car on a dual carriageway with many hazardous traffic situations, including inter-weaving and lane switching. Experts also covered a larger area on both the horizontal and vertical axes when traffic situations became more complex, whereas novices did/were not able to change their eye-movement behaviour in reaction to alternating traffic circumstances.

The domain in which eye movements is most often used to investigate the role of expertise in dynamic settings is sports. In an excellent survey article on eye-movement studies in this domain, a meta-analysis on 42 expertise studies (including 388 effect sizes) considering both dynamic (field and video) and static stimuli was performed [27]. It showed that in every single task, sport experts were better and quicker in responding than novices, and the more difficult the task (e.g., anticipating the opponents’ intentions is more difficult than recalling the location of players in a display), the more exacerbated was the difference. Moreover, when exposed to dynamic scenes, experts needed fewer fixations and these fixations were, on average, of longer duration. According to the authors, these eye-movement patterns support the interpretation that sport experts extract more task-relevant information from each fixation than novices, and that this is a sensible strategy, as it allows for more time to process task-relevant cues by virtue of minimizing the impact of saccadic suppression (i.e., the phenomenon whereby information cannot be extracted during saccades, see [23]). However, as noted above, in complex, dynamic traffic situations, experts reverted to more fixations of a shorter duration in comparison to novices [25], [26]. Additionally, here it is argued that this eye-movement pattern allows experts to sample more information from the scene on the roads by virtue of covering a larger area, a strategy yet unfamiliar to novices (see also [28]). Taken together, one may conclude that eye-movement behaviour in dynamic situations is dependent on the context, especially the domain, and the difficulty of the task and visual scene.

Until now, four eye-movement studies on the role of expertise in dynamic medical settings have been conducted – three of them in laparoscopy [16], [29], [30] and one in paediatric neurology [31]. The laparoscopy studies showed that experts spent less time on tracking the laparoscopic gear and more time fixating target locations than novices when performing computer-based simulation tasks [29], [30]. In addition, experts made fewer saccades and had a tendency for longer gaze durations during task performance [16]. The paediatric neurology study showed that experienced clinicians were more accurate in their diagnosis (seizures or disorders imitating seizures) than novices and that the time spent on relevant areas to make a correct diagnosis increased as a function of experience [31].

### The Current Study

The current study is, to our knowledge, the first eye-movement study on the role of expertise in radiology using dynamic stimuli. We set out to investigate how the performance of radiologists is reflected in eye-movement behaviour, and whether possible differences between them and radiographers and naïve participants increase with increasing task difficulty. Naturally, the radiologists represent the experts in the domain of CT examinations. The CT radiographers do not perform examinations, but look at the image quality, contrast, delineation and so on of the CT images. In principle, radiographers do not pay much attention to possible pathology, but it is not unlikely that they will recognize visually salient lesions (e.g., large liver cysts), as these lesions are easy to spot and they must have encountered such lesions during their working life on a regular basis. Given this, and given their familiarity with CT images in general, we will refer to them as semi-experts. Note that the term semi-expert is used in relationship to the tasks in the current study; they were experts in terms of the tasks they have to perform on a daily basis as radiographers. The psychology students had never examined CT images for diagnostic or technical reasons and could therefore be considered as naïve participants.

The visual stimuli were volumes of multi-slice stack-view axial CT-images with a slice thickness of 1 mm from the upper to the lower part of the abdominal area presented at different framerates, making a video-like impression (and therefore hereafter referred to as ‘videos’). CT has become a standard imaging modality for deep anatomical structures and is frequently used to detect or exclude pathologic changes. In real life, radiologists do not view CT scans with a fixed framerate and direction, but go through successive CT images manually and pause, scroll back or enlarge certain areas if that is deemed to be necessary. In this way, it is possible, for instance, to investigate one organ at a time without taking a risk that other parts of the images are left unanalysed. Being deprived of these possibilities is therefore unnatural to some extent and puts radiologists under pressure, making the task more complex than in clinical settings. At the same time, it is known that radiologists can recognize abnormalities rapidly [3], [5], [11], [12], [13] and we expect that a dynamic presentation of CT images will still allow them to detect abnormalities, especially when these abnormalities appear on a number of subsequent images. It is even possible that *radiographers* will observe abnormalities that are sufficiently salient and present on several subsequent images. However, it can be expected that in comparison to clinical settings expert performance will be compromised to some extent.

To assess the role of task difficulty directly, we manipulated the framerate and the subtlety of the abnormality. The framerate was set at three different speeds: 7, 14 and 28 images per second. The subtlety of the abnormality was manipulated by presenting the participants with videos for which they had to determine whether ELNs (the ELN task) were present, while at the same time they were asked to look for visually more salient, general visceral abnormalities (the ABN task). For the detection of ELNs, it is important to realize that lymph nodes in the abdominal area appear predominantly in the retroperitoneal space anterior to the spine and close to the aorta. The other abnormalities could appear anywhere (e.g., on the liver, the kidneys, ovaries etc.). It should be emphasized that the participants had to perform the two tasks simultaneously and that the three videos in which ELNs were present also contained other abnormalities. In fact, ELNs are very often a symptom of other pathological processes in the body and detecting them triggers the radiologist to search for the underlying causes (diseases). Nevertheless, since searching for ELNs and searching for other abnormalities are perceptually quite distinct tasks, we may claim that all participants were involved in a dual-target search [32]; that is, they constantly had to look for two visually distinct targets (in some cases ELNs appeared simultaneously with other abnormalities).

In terms of task performance, it can be hypothesized that the radiologists would outperform both the CT radiographers and the psychology students in the ELN task (**Hypothesis 1**), as the detection of ELNs is visually demanding and requires specific knowledge. For the ABN task, it can be hypothesized that both radiologists and radiographers would outperform the students (**Hypothesis 2**), as the lesions are more salient and radiographers will probably also have developed an understanding that such deviations constitute abnormalities. Given that faster presentation speed will force participants to detect and decide upon perturbations more rapidly, we also predict that higher framerates would be less appreciated by the participants and would lead to lower scores in either task (**Hypothesis 3**).

In addition to performance outcomes, we predict that the number of fixations on the relevant areas to detect lymph nodes will be larger for experts (i.e., radiologists) than for the two other participant groups (**Hypothesis 4a**). In contrast, we do not expect a larger number of fixations on other abnormalities for experts than for the other participant groups, given that the abnormalities are visually salient and likely to attract attention independent from the level of expertise. Perhaps one may even expect fewer fixations on these areas for experts, as they may recognize them more often through parafoveal/peripheral vision or they then may reach the conclusion more rapidly that the perturbation is a true abnormality (**Hypothesis 4b**).

Dynamic studies showed mixed results with respect to average fixation durations. In sports, experts reverted to fixations of longer duration [27], whereas in traffic, experts reverted to shorter fixation durations when confronted with complex dynamic scenes [25], [26]. This implies that fixation duration is a domain- or even a task-specific measure. It is therefore hard to make a general prediction, but as a working hypothesis, we follow the general assumption of Gegenfurther and colleagues [19], which holds that experts spend less time on any given location than non-experts by virtue of the experts’ more rapid extraction of domain-related information. In other words, we hypothesize that the average fixation duration will be shorter for experts than for the other two groups (**Hypothesis 5**). Our final hypothesis is related to the enlarged perceptual span experts supposedly have in comparison to non-experts. If this is not only the case in static settings [9] but also in more dynamic settings, one would expect larger average saccade amplitudes for experts than for non-experts (**Hypothesis 6**). To anticipate, we will show in the result section that the eye-movement behaviour of experts is much more adaptive than the final two hypotheses would imply.

## Methods

### Participants

Twenty-two psychology students of Turku University, 9 CT radiographers and 7 radiologists took part in the experiment. The students were naïve with respect to the topic and the task and functioned as a control group. The radiologists were senior radiologists from various Nordic countries who, on average, read about 15 CT examinations per week. They participated in the experiment during the Nordic Congress of Radiology in 2011. Five radiographers also participated in the experiment during this congress. Four other radiographers were recruited from the Turku University Hospital and were tested in the hospital itself. The radiographers indicated that they evaluated CT scans on at least a weekly basis for image quality, but that they had no diagnostic experience. All participants were healthy adults with normal or corrected-to-normal vision. The participants took part in this study voluntarily. They were informed about the purpose of the study, and they were told that they could withdraw and terminate their participation without any consequences at any time during the experiment. All participants provided their consent before experimentation began.

### Apparatus

Eye movements were recorded with a remote desktop model of EyeLink 1000 manufactured by SR Research Ltd; the recording was monocular. The eyetracker is an infrared video-based tracking system with hyperacuity image processing and a spatial resolution of 0.4 degrees. An infrared LED for illuminating the eye was positioned next to the eye-movement camera. A chin-and-forehead rest was used to minimize head movement. The videos were presented on a 21.3-inch EIZO RadiForce MX210 monitor with a refresh rate of 60 Hz. The monitor is designed for radiology work stations and is ideal for viewing CT medical images. Participants were seated 70 cm in front of the monitor.

### Materials

The experiment contained 1 practice and 9 target videos containing approximately 600 cross-sectional, multi-slice stack-view CT images from the upper to the lower part of the abdomen (more specifically, from the diaphragm to the pelvic floor). The abdominal CT scans were performed by a Siemens Somatom Sensation 64 scanner (Siemens, Erlangen, Germany). The images were obtained after intravenous contrast enhancement (iohexol Omnipaque, Nycomed Imaging 350 mg iodine/mL, dose 1 mL/kg at an injection rate of 2 mL/second) in the venous phase. The reconstructed images were continuous images with a slice thickness of 1 mm and slice increment of 0.7 mm. The images were shown using standard abdominal window and level settings (level 40, window 360).

There were three types of videos: videos including visceral abnormalities such as liver cysts (ABN videos); videos including ELNs in addition to visceral abnormalities (ELN videos); and videos without any abnormalities (NORM videos).

**Figure 1:**
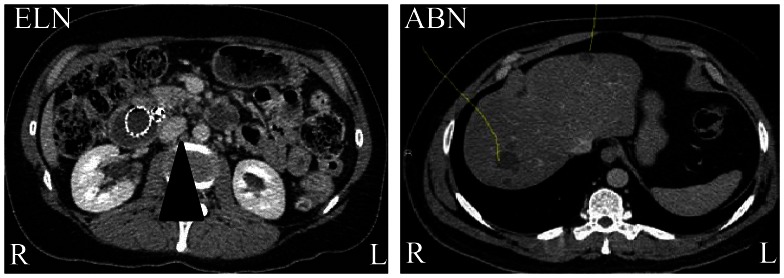
Cross-sectional abdominal CT-images used in the experiment. The CT-images depict an enlarged lymph node from an ELN-video (arrowhead in a) and liver cysts from an ABN-video (yellow lines in b). Note that the left and right side of the body are switched in the images.

A picture of the two former video types is presented in Figure 1. The videos were presented at different framerates, either at 7 i/s, 14 i/s or 28 i/s (i/s stands for images per second). More specific information on the patients depicted in the videos can be found in Table 1.

**Table 1:** Patient information for each video used in the experiment.

Video	Type	Gender Patient	Age Patient	Abnormality	Nr. of Images
N8	norm	female	40	–	510
N1	norm	male	19	–	669
N3	norm	male	37	–	580
N9	norm	female	48	–	617
N4	abn	male	49	liver cysts	591
N5	abn	male	44	dilated upper urinary tract	677
N7	abn	female	37	ovarian cysts	542
P1	eln	male	46	colon & bladder tumor; fluid in abdominal cavity around liver	625
P2	eln	male	30	ascites; fluid in abdominal cavity; rectal tumor	723
P3	eln	female	45	cirrhosis	512

### Procedure

Prior to the experiment, participants were instructed that they would be exposed to 10 abdominal CT-image videos, the first one being a practice video. Their task was to detect ELNs and/or other visceral abnormalities. They were also informed that some videos did not contain any abnormalities and that they were not supposed to report skeletal abnormalities. Finally, they were informed that they would have to answer questions after each video about the presence of ELNs and other abnormalities, and about the framerate of the video.

Each participant saw a NORM video as a practice trial and, subsequently, three videos of each video type. The first three experimental videos were presented at a framerate of 7 i/s, the next three at 14 i/s and the final three videos at 28 i/s. Each participant saw each type of video (ABN, ELN and NORM videos) in each framerate (7 i/s, 14 i/s and 28 i/s), even though every individual video appeared in only one framerate during an experimental session. Materials were counterbalanced such that videos occurred at different framerates across participants and care was taken that each video type appeared at each framerate to an equal degree across participant groups.

Before the experimental session, the eye tracker was calibrated using a 9-point calibration grid that extended over the whole computer screen. Prior to each video, the calibration was checked by presenting a fixation point in the centre of the screen; if needed, calibration was automatically corrected, after which the video was presented. The four questions that had to be answered after each video were: (1) Did you encounter ELNs? (answer: yes/no); (2) How sure are you of your answer to question 1? (answer: 0%–100%); (3) Did you encounter other soft-tissue abnormalities? (answer: yes/no); and (4) What did you think of the framerate? (answer: too slow, adequate, too fast). For questions (2) and (4), participants had to mouse click a line to respectively indicate how sure they were and how much they appreciated the framerate. After the practice video, presented at framerate 7 (i.e., 7 i/s), three experimental videos with the same framerate (7) were presented followed by three videos at framerate 14 and finally three videos at framerate 28. After the experiment, each participant was interviewed about his or her general experience with CT scans and radiologists were asked how many CT-scan examinations they performed per week. Participants did not get feedback about their responses after each trial separately, but were informed about their performance at a later stage (some right after the experiment, some by email a week later).

## Results and Discussion

### Performance (Hypotheses 1 and 2)

For both ELNs and other abnormalities (ABN), we calculated the number of true positives (an indication that the ELN or ABN was present in the video when either was indeed present, TP) and true negatives (an indication that the ELN or ABN was absent in the video when either was indeed not present, TN). Table 2 lists the outcomes for the two response types as well as the overall accuracy for each participant group.

**Table 2:** Mean performance on ELN and ABN detection across the three expertise groups.

Level of Expertise	Task	TP (%)	TN (%)	Accuracy (%)
Naïve	ELN detection	52	49	50
Semi-experts	ELN detection	44	59	54
Experts	ELN detection	71	90	84
Naïve	ABN detection	36	73	50
Semi-experts	ABN detection	81	72	78
Experts	ABN detection	86	57	74

With respect to ELN detection and in support of Hypothesis 1, experts outperformed the other two groups, both in detecting ELNs when they were there (TP) and in indicating that they were not there when they were absent (TN). This led to a significant group effect in the ANOVA analysis for overall accuracy, *F*(2, 35) = 7.64, *p*<.01, with post-hoc comparisons (using Tukey’s honestly significant difference (HSD) test) showing that experts performed better than both semi-experts and naïve participants (both *p*s<.02). Perhaps somewhat surprisingly, there was no difference in ELN detection between the semi-expert and naïve participants (p>.2), with both groups performing at chance level. In line with these findings, the answers to the second question indicated that experts were much more confident about the presence or absence of ELNs in the videos than semi-experts, who, in turn, were more confident about their answers than the naïve participant group (despite the fact that they did not perform better in the ELN task). More specifically, when dividing the line up into three areas (left: unsure; middle: reasonably sure; right: very sure), naïve participants mostly clicked the line on the left side (L: 57%; M: 35%; R: 8%), semi-experts in the middle (L: 32%; M: 54%; R: 14%) and experts on the right side (L: 3%; M: 36%; R: 61%). Chi-square tests with Yates’ continuity correction showed that both the group effect and all pairwise comparisons were highly significant (all *p*s<.01).

With respect to ABN detection, a significant group effect emerged for overall accuracy *F*(2, 35) = 22.54, *p*<.01. In line with Hypothesis 2, experts (E) and semi-experts (SE) performed better than naïve participants (N) did (both *p*s<.001). The difference could be completely ascribed to the failure of naïve participants to observe abnormalities (for both SE vs. N and E vs. N, *p*s<.001) rather than failing to indicate that there were no abnormalities when they were absent (*F*<1). In general, this shows that naïve participants tended to indicate not having observed any abnormalities, regardless of whether they were there or not. There was no difference between experts and semi-experts (p>.2). The performance results confirmed that the division of the participants into experts, semi-experts and naïve participants was warranted.

With respect to the individual videos, it is noticeable that experts had a harder time in detecting ELNs in video P1 (detected by 3/7 radiologists, detection rate 43%) than in the other two ELN videos (86% detection rate). This may be caused by the fact that in video P1 the largest ELN was not located in the retroperitoneum near the spine (where the majority of lymph nodes in the abdominal area are located), but in the mesentery of the right lower abdomen. Other ELNs anterior to the spine did appear in P1, but they were visible for a relatively short period only (20 images, amounting to 1 to 3 seconds, depending on the framerate). We may conclude that, even for experts, subtle perturbations are hard to detect when they are in unexpected locations or when they cannot be inspected for a longer time. Additionally, with respect to the detection of other abnormalities, one video stands out. More precisely, in 5 of the 6 ABN videos the large majority of the experts and semi-experts detected the visceral abnormality (75%, 88%, 94%, 94%, 94% and 94%, respectively), but for one video (N7), the detection rate of the abnormality (ovarian cyst) was only 56%. On closer inspection, the ovarian cyst in video N7 was more difficult to detect than other abnormalities in other videos due to lower visual saliency (more background noise) and a fewer number of images where it was present (24).

### The Effect and Appreciation of Framerate (Hypothesis 3)

Table 3 summarizes appreciation and accuracy scores as a function of framerate. With respect to appreciation scores, we divided the response line on the framerate question (‘What did you think of the framerate?’) into three equal parts. Mouse clicks on the left side, middle or right side indicated that the framerate was too slow, was adequate or was too fast, respectively. The results showed that both framerate 7 and 14 were considered to be adequate by all participant groups. In other words, all participants were quite comfortable with the slower framerates, despite the unnatural, forward machine-paced presentation style. For all participant groups, framerate 28 elicited ‘too fast’ responses most often (for all *χ* ^2^-tests, global and per participant group, *p*s<.01). It is notable that naïve participants were the participants that most often indicated the 28 i/s framerate to be adequate. Possibly they were less bothered by the fast framerate due to their lack of experience or skill, which made it difficult to detect lesions at any rate. The other two participant groups clearly judged the 28 i/s framerate to be too fast. To our minds, this indicates that they were engaged in the tasks and that they could not appreciate them when images were sequenced too rapidly for proper evaluation. Framerate 7 and 14 were equally well appreciated (*p*s>.10). Surprisingly, framerate did not affect performance (see Table 3), as in neither task was there evidence for a Framerate × Expertise interaction or a main effect of framerate (all *p*s>.2). More specifically, semi-experts and experts performed equally well in the ABN task under high, medium and low framerate circumstances. Moreover, experts managed to get 86% of the cases correct in the visually demanding ELN task, even though videos were presented at framerate 28.

**Table 3:** Mean performance and appreciation of videos as a function of framerate.

Level of Expertise	Framerate (in i/s)	Appreciation (in %)			Accuracy ABNTask (in %)	Accuracy ELNTask (in %)
		Too Slow	Adequate	Too Fast		
Naïve	7	8	92	0	42	53
	14	0	92	8	48	44
	28	0	53	47	61	53
Semi-experts	7	17	75	8	75	61
	14	4	70	26	74	51
	28	0	26	74	85	48
Experts	7	19	59	22	70	81
	14	0	62	38	71	86
	28	0	29	71	81	86

### Fixations on Relevant and Irrelevant Areas (Hypothesis 4)

**Figure 2:**
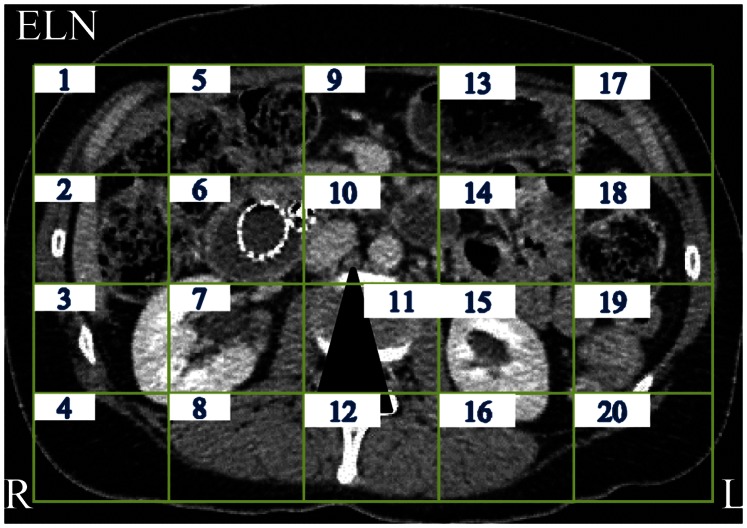
Cross-sectional abdominal CT-image with an ELN divided into 20 equal-sized areas of interest (AOIs). The enlarged lymph node in this image is pointed out by the black arrowhead and resides in Area 10, the area where most lymph nodes are located in all images across videos.

We divided the CT images into 20 areas of interest (see Figure 2) and determined the number of fixations for each participant group per area. For the ELN task, the definition of a relevant area was relatively easy. That is, as mentioned earlier, most lymph nodes in the abdominal area are located in the retroperitoneum anterior to the spine, corresponding to Area 10 in the CT images. As awareness about this should grow with increasing expertise, we predicted that the number of fixations in Area 10 would be larger for experts than for the other two groups (Hypothesis 4a).

**Figure 3:**
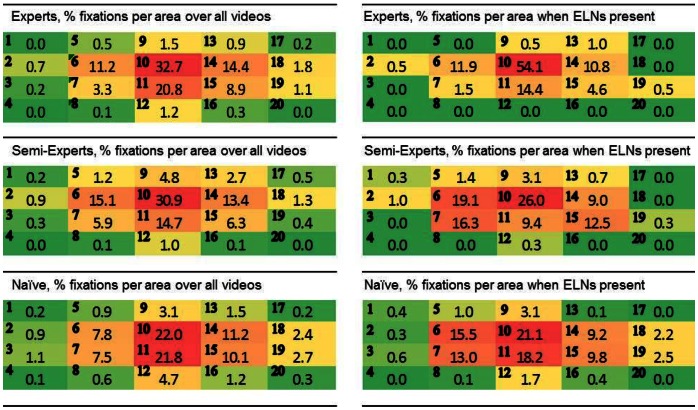
Heatmaps of the distribution of fixations over all 20 areas for each participant group. The left panels include all fixations over all videos; the right panels depict the fixation distributions for images in ELN videos where enlarged lymph nodes are present.

The left panels of Figure 3 are heat maps depicting the percentage of all fixations over all videos for each participant group. In line with the hypothesis, Area 10 is relatively more often fixated by experts than by naïve participants, F(2, 35) = 7.04, *p*<.01; E vs. N, p<.01). Against the hypothesis, semi-experts also dedicated relatively more fixations to this area than did naïve participants (SE vs. N, p<.03) and there was no difference between experts and semi-experts (*p*>.4). Subsequently, we performed a separate ANOVA for ELN videos to assess whether there was an increase of fixations in Area 10 on images where lymph nodes were actually enlarged in comparison to those where they were not. We coined this factor *ELN_Present.* We excluded video P1 from this analysis, because the number of experts detecting ELNs in this video was below 50%. The 3×2 ANOVA showed an interaction between *Expertise* and *ELN_Present*, *F*(2, 35) = 8.94, *p* = .01. Separate *t*-tests for each participant group showed that experts significantly increased the relative number of fixations in Area 10 on images where ELNs were present (from 30% up to 54%, *t*(6) = 6.34, *p* = .001) or the amount of time for that matter (from 35% to 60%), but the other participant groups did not (*t*s<.1). Taken together, it can be concluded that both semi-experts and experts knew where to look for lymph nodes, but only experts reverted to careful foveal inspection by increasing the number of fixations in the relevant area when lymph nodes turned out to be enlarged.

**Figure 4:**
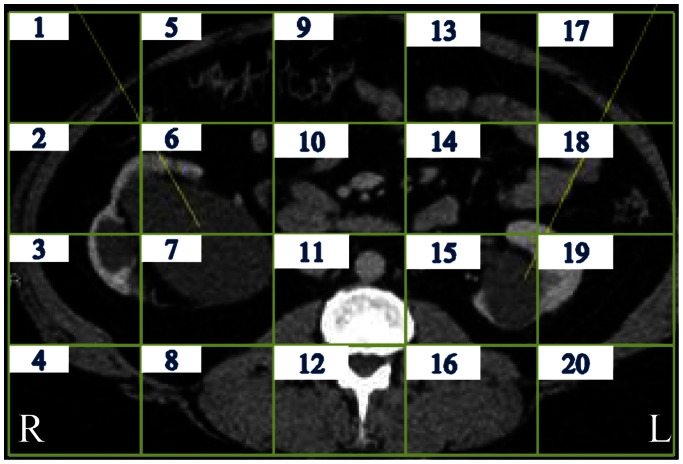
Cross-sectional abdominal CT-image depicting hydronephrosis. The hydronephrosis is present at the right side in area 2, 3, 6, and 7 and on the left side in area 15.

With respect to other abnormalities, we could not determine beforehand what areas were relevant, as the abnormalities could, and did appear in different organs. However, we could also assess here how frequently the eyes fixated on areas when the abnormality was present in comparison to when it was absent. Unfortunately, the only video that could be considered for this analysis was video N5 including hydronephrosis or – in other words – a dilated upper urinary tract; this dilated tract occupied areas 2, 3, 6 and 7 of the kidney on the left side and area 15 of the kidney on the right side (see Figure 4). In video N7 the abnormality (ovarian cyst) was only present for a short time and often not detected and video N4 included cysts all over the liver and, as the liver occupies at its largest more than 50% of the image (from area 1 to 11), the relevant area would be too large. In other words, video N5 was the only video with a fairly local abnormality that could contribute to the current analysis. The area-of-interest (AOI) analysis for this video nevertheless gave a good insight into the different strategies of each participant group when salient abnormalities appeared.

Table 4 lists the percentage of fixations dedicated to areas 2, 3, 6, 7 and 15 when hydronephrosis was visible (from image 190 to 390) and the percentage of fixations in these areas when it was not there (images 1–189 plus images 391–679). The global 3×2 ANOVA again shows a significant interaction between the participant group and the presence of abnormality, *F*(2, 35) = 5.51, *p*<.01. This time, both semi-experts and naïve participants increased the relative number of fixations on the relevant areas, with both *p*s<.01, but the increase was larger for semi-experts than for naïve participants (as supported by the interaction between the group and abnormality presence in a 2×2 analysis excluding the experts, *F*(1, 29) = 6.44, *p*<.02). Experts did not increase the number of fixations in the presence of the abnormality, *t*>1. It is noticeable that even though experts did not fixate the relevant areas as much as the other two participant groups when abnormalities were present, their performance was not compromised; that is, all 7 experts detected the abnormality (see Table 4). The abnormality was observed by 8 out of 9 semi-experts and 8 out of 22 naïve participants as well.

**Table 4:** Percentage fixations on area 2, 3, 6, 7, and 15 with and without hydronephrosis.

Level of Expertise	% Fixations without Hydronephrosis Present	% Fixations with Hydronephrosis Present	% Correct
Naïve	22.3%	29.7%	36%
Semi-experts	20.7%	40.1%	89%
Experts	20.9%	23.4%	100%

### Statistical Considerations for Average Fixation duration and Saccadic Amplitude Analyses

Typically, analyses on average fixation duration and saccadic amplitude in medical-image perception studies have been performed by ANOVAs with separate participant (*F*1) and (sometimes) item (*F*2) analyses. As most studies only include a small number of experts and typically also a small number of items, the analyses based on average participant and item scores are typically compromised by low statistical power. In this study, we make use of statistically more powerful linear mixed-effects (LME) models to model all fixations and saccades generated by the 38 participants, eliminating the need of a priori averaging over participants and items. In the mixed-effects multiple-regression models, we included participants and videos as crossed random effects, allowing us to explore simultaneously our predictors of interest and their interactions as fixed-effect factors, while accounting for between-participants and between-videos variance [33]. Only those fixed effects that reached significance at the 5% level in a stepwise, backward-elimination procedure using the model comparison likelihood ratio test are reported and presented in the respective tables of Appendix S1. The random effects included in our models significantly improved the explanatory value of those models, as indicated by significantly higher values of the maximum likelihood estimate of the model with a given random effect compared to the model without that random effect (all *p*s<0.0001 using likelihood ratio tests; for detailed treatment of random effects in mixed-effects models, see [34]).

Specifications for all models reported in Appendix S1 present the output of the pvals.fnc() function in library languageR of R statistical software [35]. The specifications include estimates of the regression coefficients; highest posterior-density intervals (HPDs), which are a Bayesian measure of confidence intervals; *p*-values estimated by the Monte Carlo Markov chain (MCMC) method using 10,000 samples; and *p*-values obtained with the *t*-test for fixed effects using the difference between the number of observations and the number of fixed effects as the upper bound for the degrees of freedom (for the detailed treatment of the method, see [33], [34], [36]). For the effects reported in the body of the paper we provide *p*-values estimated by the MCMC method using 10,000 samples.

The fixed factors entered into the model were the ones that were directly manipulated, namely:

- Level of Expertise, *Expertise*, including 3 levels: Expert (E); Semi-Expert (SE); Naïve (N).
- Video type, *Video*, including 3 levels: Normal (Norm); With visceral abnormalities (ABN); With ELNs and visceral abnormalities (ELN).
- Framerate, *FRate*, including 3 levels (1 slow, 7 i/s; 2 medium, 14 i/s; 3 fast, 28 i/s).
- In addition to the main effects, we entered both the interactions between *Expertise* and *Video* and the interaction between *Expertise* and *FRate*.

We excluded one ELN video (P1) and one ABN video (N7) because – as mentioned above – they generated many incorrect responses from experts and semi-experts as a result of the limited number of images where the perturbations were visible. The other 7 videos generated 32,671 fixations altogether (after merging close-by fixations with at least one of them being smaller than 50 ms). All models are fully presented in Appendix S1, significant results are mentioned in the running text and interactions are depicted by figures.

### Average Fixation Duration

Raw fixation durations ran from 50 to 2500 ms and were not normally distributed. Since a normal distribution is a requirement for the LME models, we normalized the distribution by a logarithmic transformation of the fixation duration values. Further normalization was achieved by removing data points that fell outside the range of −2.5 to 2.5 *SD* of the mean log-fixation duration. After these removals, 31,950 fixations were left.

The model (see Table A in Appendix S1) showed significantly longer average fixation durations for the videos presented at a framerate of 28 i/s than for videos presented at the other two framerates (7∶361 ms; 14∶358 ms; 28∶375 ms; *p*s<.001). There was no interaction between *FRate* and *Expertise*, so all participant groups considered it to be a sensible strategy to stay fixated longer on any given location when information floated by very rapidly.

**Figure 5:**
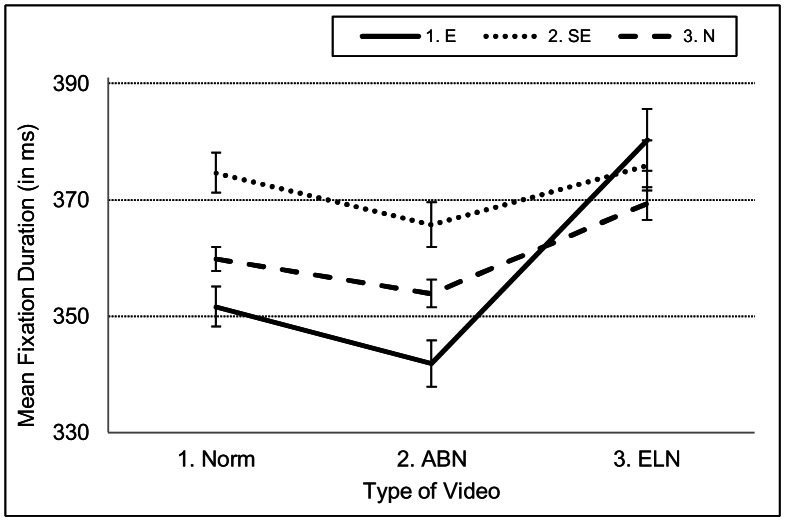
Interaction between *Expertise* and *Video* for average fixation duration. The figure depicts that experts reverted to longer fixation durations in ELN-videos than in other videos, whereas semi-experts and naïve participants did not.

Hypothesis 5 holds that average fixation duration is shorter for experts than the other two groups by virtue of domain-related information being more rapidly extracted by experts than by non-experts [19]. However, there was no significant main effect for *Expertise*, even though experts had numerically shorter average fixation durations than non-experts (E: 356 ms; SE: 372 ms; N: 360 ms). Importantly though, there was a significant interaction between *Expertise* and *Video* (*p*<.05), see Table A in Appendix S1. This interaction (depicted in Figure 5) reflects that experts have longer average fixation durations for videos that contain ELNs in comparison to other video types, whereas this is not the case for other participant groups; that is, average fixation duration is quite stable across video types for semi-experts and naïve participants.

### Average Saccade Amplitude

Hypothesis 6 holds that experts will make longer saccades than non-experts by virtue of their enlarged perceptual span. As for fixation duration, we modelled saccadic amplitude using mixed-effects multiple-regression models with random intercepts for participant and video. Given the variability in saccadic amplitudes, a simple logarithmic transformation did not yield a normal distribution. To obtain a normal distribution, we first excluded extreme outliers from the dataset. Making a saccade from one side of the video to the other side amounts to about 10 degrees of saccadic amplitude and can be considered as normal saccadic behaviour. Saccades with amplitudes larger than 11 degrees were saccades preceded or followed by a fixation outside the video area and can be considered as extreme, so all such saccades were excluded before analyses. After these removals, 31,998 saccades were left. Subsequently, we calculated the power transformation for normalization by making use of the powerTransform function in the R-package car. The estimated power transformation was an exponentiation of 0.3217, so all saccadic amplitude values were submitted to this transformation before modelling. The exponential transformation and the exclusion of outliers rendered the final residuals of the model normally distributed.

**Figure 6:**
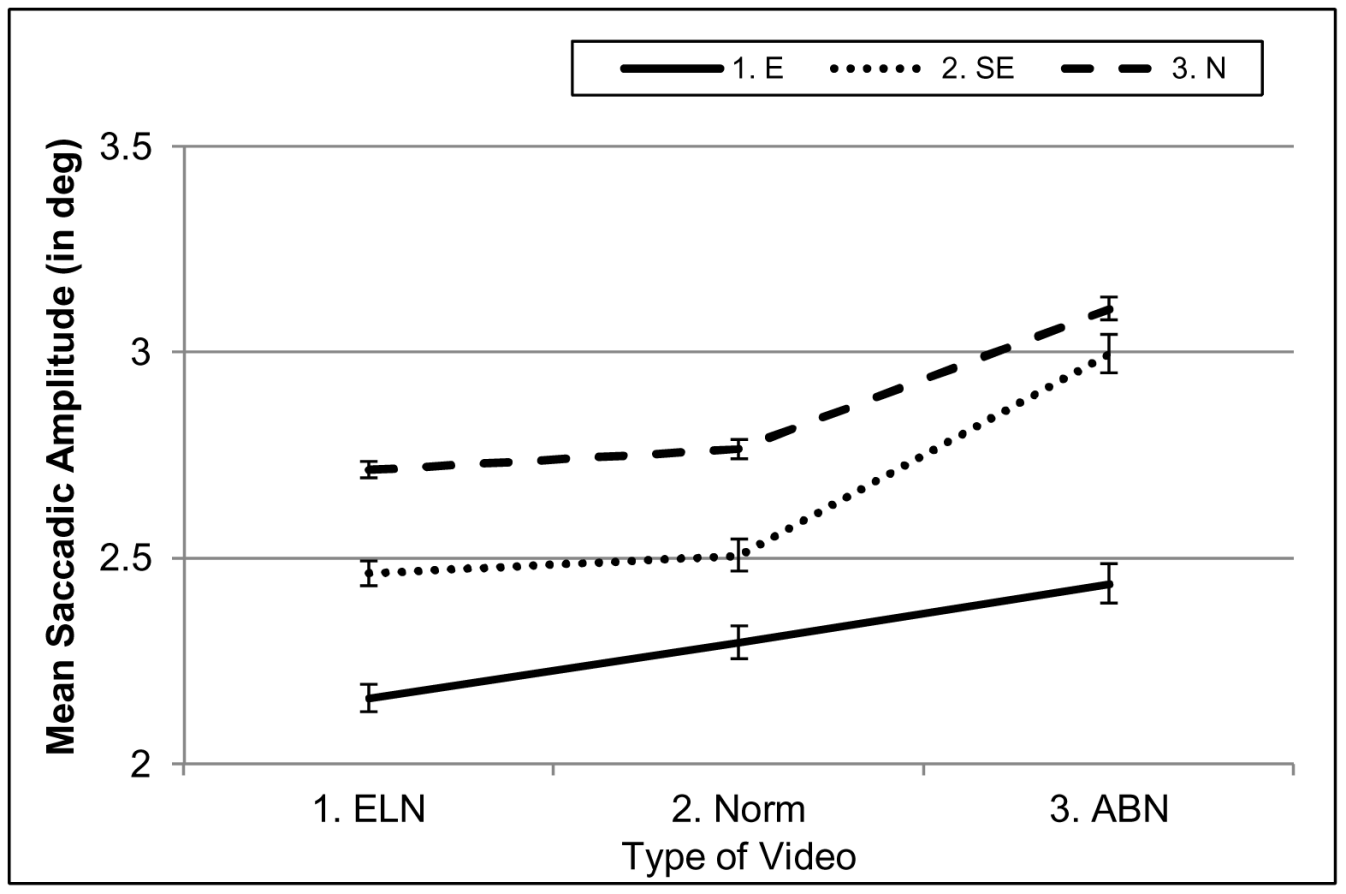
Interaction between *Expertise* and *Video* for saccadic amplitude. The figure depicts that semi-experts and to some extent naïve participants used longer saccades in ABN-videos than in other videos, whereas experts did not.

Instead of larger saccadic amplitudes for experts, amplitudes were significantly shorter for experts than for other groups (E: 2.31; SE: 2.64; N: 2.86; *p*<.05). There was also an interaction between *Expertise* and *Video* (see Figure 6 & Table B in Appendix S1, *p*<.05). The interaction reflects that semi-experts had longer saccadic amplitudes in ABN videos than in other videos, whereas experts had not. Naïve participants, in turn, made larger saccades in ABN videos than in other videos, but the difference in saccadic amplitude between them was not as large as that of the semi-experts. This is in line with the AOI analyses, which showed that semi-experts more frequently inspected abnormalities than naïve participants, who, in turn, more frequently inspected them than did experts. Larger saccades can be linked to the inspection of abnormalities at different locations, which fits the profile of the two videos included in the ABN conditions (N4, with cysts at different locations on the liver and N5 with hydronephrosis on the right and left side).

### Local Analyses

The analyses presented above revealed how different participant groups responded with their eye-movement behaviour to different types of videos. Yet it remains unclear as to whether measures such as fixation duration and saccadic amplitude also fluctuate as a function of the visual scene within videos. That is, it would not be unlikely that eye-movement behaviour is different in sections of videos where ELNs or general abnormalities are actually present in comparison to those sections where they are not. To investigate this issue, we conducted separate analyses for the feasible ELN videos (P2 and P3) and ABN videos (N4 and N5). In both cases, we used the same normalization procedures for fixation duration and saccadic amplitude as in the global analyses.

For the ELN videos, we entered the following factors into the LME models: *Expertise* (E; SE; N), *FRate* (7 i/s; 14 i/s; 28 i/s) and the presence of an ELN, (*ELN*: Yes; No). We were mostly interested in the impact of the factor *ELN* and how it interacted with *Expertise*. For *average fixation duration*, experts – unlike the other participant groups – had numerically longer fixation durations in the presence of ELNs, but this did not result in a significant interaction between *Expertise* and *ELN* (*p*>.2).

**Figure 7:**
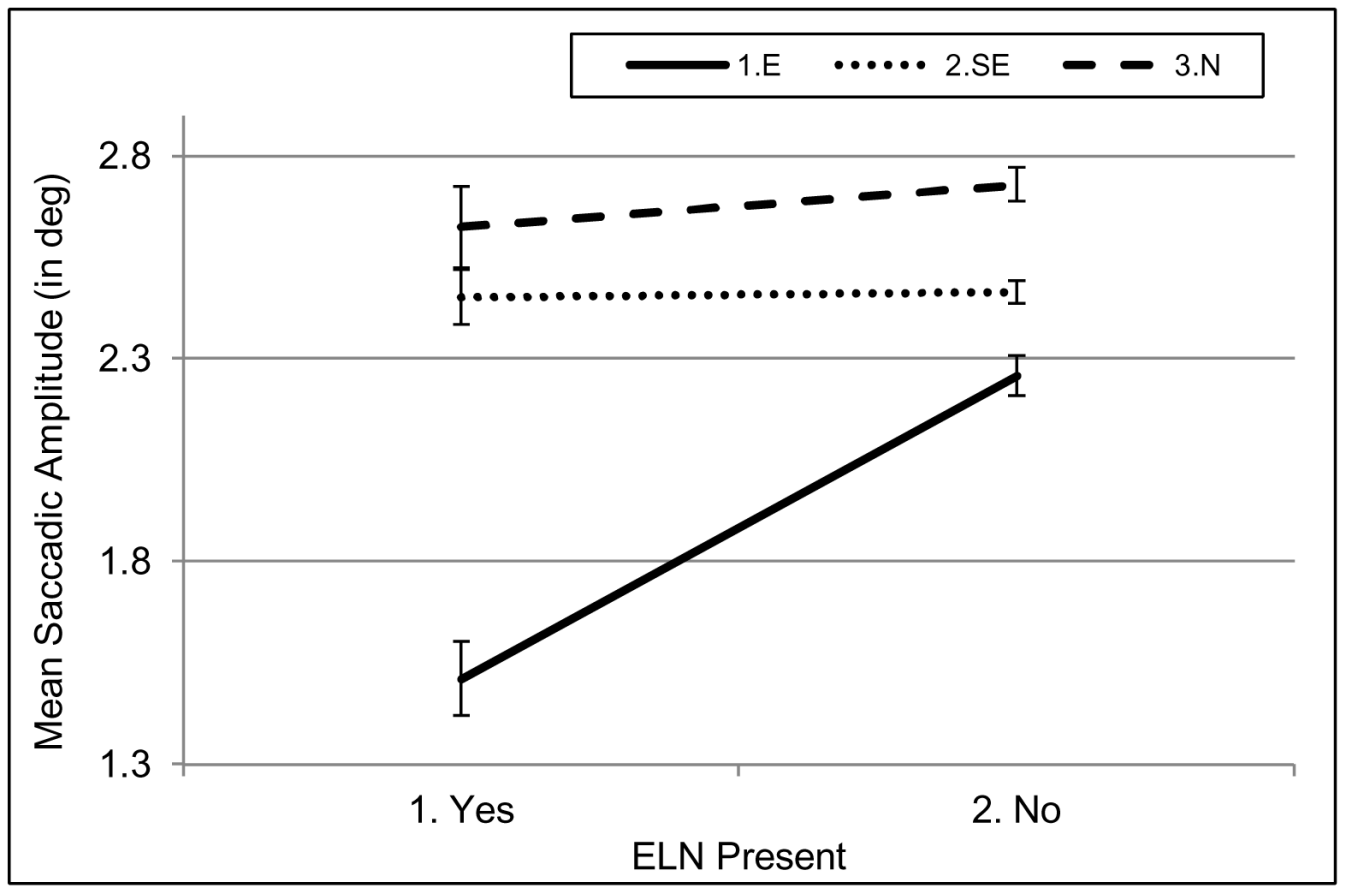
Interaction between *Expertise* and *Presence of ELN* for saccadic amplitude in ELN-videos. The figure depicts that unlike the other groups, experts made shorter saccades in video sections where enlarged lymph nodes were present than in sections where they were absent.

In contrast, the interaction between *Expertise* and *ELN* was highly significant for *saccadic amplitude* (see Table C in Appendix S1). The interaction reflects that experts reverted to much shorter saccades in those sections of the videos where ELNs were present. In these sections, experts made saccades of on average 1.51^o^, whereas their saccadic amplitude was on average 2.26^ o^ when ELNs were not present. As can be derived from Figure 7, semi-experts and naïve participants did not adjust their saccadic amplitude as a function of the presence of ELNs.

**Figure 8:**
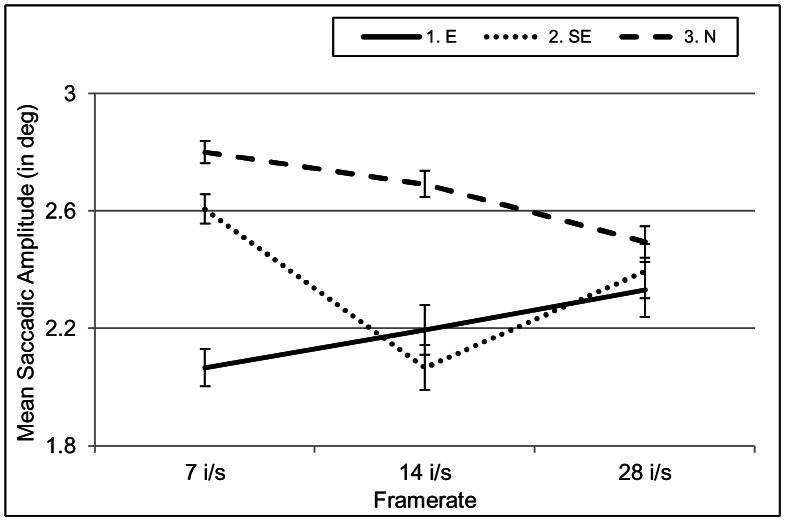
Interaction between *Expertise* and *Framerate* for saccadic amplitude in ELN videos. Experts made progressively longer and naïve participants progressively shorter saccades as a function of increasing framerate. Semi-experts behaved like naïve participants in the lowest framerate and like experts in the higher framerates.

Table C in Appendix S1 and Figure 8 also show that there was an interaction between *Expertise* and a framerate of 7 i/s–28 i/s. The interaction reflects that experts reverted to longer saccades, whereas naïve participants reverted to shorter saccades when the framerate increased. This change of strategy on the expert part probably reflects the attempt to extract more information from a larger area during one fixation when changes in information flow happen rapidly. Semi-experts behave like naïve participants in the slowest framerate, but like experts in the faster framerates.

For the ABN videos, we entered the following factors into the LME-models: *Expertise* (E; SE; N), *FRate* (7 i/s; 14 i/s; 28 i/s) and the presence of an ABN, (*ABN*: Yes; No). Naturally, we were mostly interested in the impact of the factor *ABN* and how this factor interacted with *Expertise*. Again, the analyses on average fixation duration did not yield a significant interaction between *ABN* and *Expertise* (*p*>.5).

**Figure 9:**
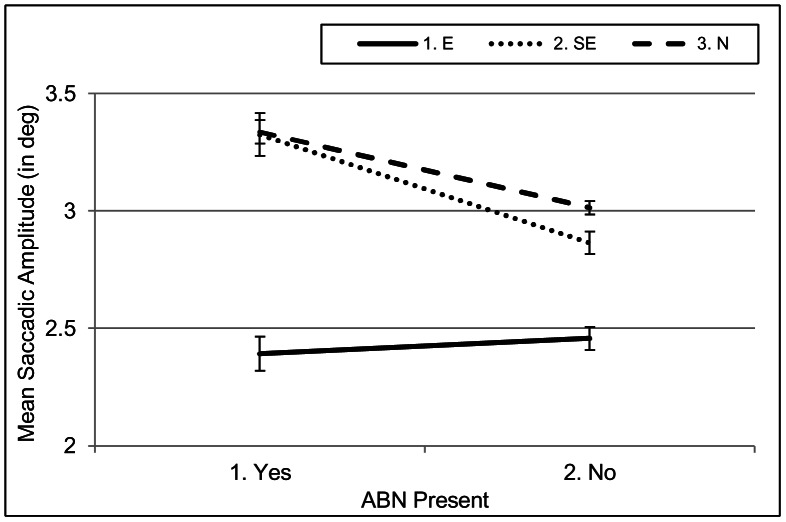
Interaction between *Expertise* and *ABN* for saccadic amplitude in ABN videos. Unlike experts, both semi-experts and naïve participants reverted to clearly longer saccadic amplitudes in video sections where abnormalities were present.

With respect to saccadic amplitude, experts made much shorter saccades than non-experts (E: 2.44; SE: 3.00; N: 3.11), a difference that was actually even more pronounced than in the global analyses. Table D in Appendix S1 and Figure 9 show that the main effect for *Expertise* was quantified by an interaction with *ABN*: Both semi-experts and naïve participants made longer saccades in those video sections where abnormalities were present in comparison to video sections where they were absent, whereas experts’ saccadic amplitudes were unaffected by the presence of abnormalities.

### General Discussion

The present eye-movement study examined how professional experience is reflected in task performance and eye-movement behaviour when viewing dynamic scenes in the domain of radiology. A group of experts, semi-experts and naïve participants were exposed to three types of CT-image videos to perform two distinct tasks simultaneously while their eye movements were registered. The first task was to determine whether the videos contained ELNs (the ELN task). This was a visually demanding task, as the ELNs were hard to discern in our videos. The second task was to determine whether the videos contained visually salient visceral abnormalities (the ABN task). In the current study, these tasks had to be performed for CT images presented at a low, medium and high framerate.

The **task-performance results** showed that – in line with **Hypothesis 1**– radiologists outperformed the other two groups in the ELN task. Their average accuracy rate amounted to 84%, whereas the other two groups performed at chance level. Moreover, in support of **Hypothesis 2**, both radiologists and radiographers performed equally well on the ABN task with a success rate of about 75%, and both groups outperformed naïve participants, who again performed at chance level. It thus seems that the radiographers were able to detect visually salient abnormalities, and therefore it can be concluded that they had some diagnostic competence. However, they overestimated themselves somewhat by indicating that they were reasonably sure about their answers in the ELN task, whereas they performed at chance level in this task. One may thus conclude that radiological training and experience is required to detect whether subtle abnormalities such as ELNs are absent or present in dynamically presented CT images.

In line with **Hypothesis 3**, the highest framerate of 28 images per second (i/s) was the least appreciated, but against this hypothesis, we found that increasing the framerate did not significantly affect accuracy rates: Radiologists performed both tasks equally well under all framerate conditions and also radiographers’ success rate in the ABN task was independent from the framerate at which the CT images were presented. In clinical practice, volumes of CT images are viewed in a free-viewing fashion with the possibility to pause, proceed very slowly or go back in the image sequence at any given time. The lower the framerate, the more the current presentation style reminds the clinical setting; that is, it may well be that under some circumstances a radiologist takes one second to go through 7 CT images with a thickness of 1 mm. However, it will be extremely rare that a radiologist evaluates 28 such images in one second. In that sense, expert performance in especially the visually demanding ELN task at this framerate can be considered remarkable, even though it ties in with other studies showing that experts are able to detect abnormalities very rapidly [3], [5], [11], [12], [13].

In the analyses, we approached differences in **eye-movement behaviour** as a function of expertise by considering three different eye-movement measures: the number of fixations in relevant areas, the average fixation duration and the average saccadic amplitude. Differences between groups appeared in all three measures. In line with **Hypothesis 4a**, our AOI analysis showed that experts dedicated relatively more fixations to the area anterior to the spine than naïve participants did. Slightly to our surprise, semi-experts dedicated an equal number of fixations to this area as experts did when the images did not contain ELNs. This implies that both experts and semi-experts knew beforehand that the majority of lymph nodes in the abdominal area are located in the retroperitoneum anterior to the spine. Both groups thus seem to use a top-down strategy in viewing the CT-image videos. When the lymph nodes anterior to the spine were de facto enlarged, experts increased the number of fixations and the time spent on this area. Semi-experts did not increase the number of fixations when ELNs appeared on the images, which is in line with their at-chance performance in the ELN task.

**Hypothesis 4b** stated that the visually salient other abnormalities would attract the same number of fixations for semi-experts and naïve participants, but perhaps slightly fewer fixations from experts. The results reported here do not fully support this hypothesis. That is, the semi-experts dedicated more fixations to the relevant areas in ABN videos than naïve participants, who, in turn, used more fixations to inspect these areas than did experts. The increased number of fixations on relevant areas by naïve participants implies that they had perceived the visual deviations, but, the accuracy score indicates that most of them did not judge these deviations to be abnormal. In contrast, all experts and almost all semi-experts did classify the deviations as abnormalities, but the striking difference in fixation number on the relevant areas between the two groups indicates that experts did not need as much visual input to classify the visually salient deviations as abnormalities.

**Hypothesis 5** held that average fixation duration is shorter for experts than for the other two groups by virtue of experts’ more rapid extraction of domain-related information. However, there was no main effect for fixation duration to support this hypothesis. What we did find was an interaction between level of expertise and video type: Unlike the other participant groups, experts increased their fixation durations when ELNs were present in the videos. The longer fixation durations in ELN videos may reveal a general roll-up-the-sleeves strategy. That is, in the ELN videos of this study, the abnormalities appeared early in the image sequence (in P2 starting from the first image, in P3 starting at image 30) and this may have prompted the radiologists to more carefully inspect the visual scene, leading to, on average, longer fixation durations.

**Hypothesis 6** predicted, on average, longer saccadic amplitudes for experts than for the other two groups. In the global analyses on saccadic amplitude, we found exactly the opposite: A main effect for expertise with experts reverting to generally shorter saccades than non-experts. The generally shorter saccades imply that they more systematically inspect smaller areas than non-experts do (especially Area 10). This is also supported by a larger number of short saccades (saccades with an amplitude of less than 1.6^o^) by experts than semi-experts and naïve participants (E: 45%; SE: 38%; N: 29%). Given that lymph nodes are typically located near each other, this seems to be a sensible strategy. The local analyses showed that when enlarged lymph glands appeared on the images, experts started to revert to even shorter saccades, indicating that they had entered a stage of focused analysis. In this stage, they probably focused their attention during a fixation on a really small area, possibly on one single lymph node at a time. It should be noted that this behaviour is very systematic across radiologists, as all seven radiologists reduced their saccadic amplitude in the presence of ELNs. Experts did not make longer or shorter saccades in ABN videos than in the other types of video. In contrast, semi-experts and naïve participants made longer saccades in ABN videos than in other video types. The local ABN-video analyses showed that the longer saccades were related to the actual presence of visceral abnormalities within these videos. Experts neither reverted to longer saccades in these videos in general, nor increased the saccadic amplitude in the presence of abnormalities. This is in line with the results of the AOI analysis and shows once more that they came more quickly to the same conclusion than the semi-experts did.

### Theoretical Implications of the Current Study

Correct decisions about the size of lymph nodes most likely requires focal [4] or selective [2] vision. Both experts and semi-experts anticipated this to be the case and dedicated a relatively high number of fixations to the area in front of the spine where lymph nodes are predominantly located. Thus, it seems that both groups were prepared to use focal/selective vision to come to the right decision about the presence or absence of ELNs. Note, however, that a large number of fixations in a relevant area are not sufficient to detect subtle lesions. That is, semi-experts did not perform above chance level in the ELN task, which is most likely due to a lack of specific conceptual knowledge and/or perceptual skills required for this task. Even for experts, the detection rate dropped when ELNs were present but located outside the pre-targeted area (video P1). Additionally, the detection of a less salient general abnormality (ovarian cyst, video N7) was more difficult than the detection of other more salient abnormalities. This in line with earlier studies showing that lesion subtlety influences detection accuracy and the visual-search behaviour of radiologists. For instance, in static scenes, subtle lesions are detected later and generate longer dwell times than obvious lesions if they are detected at all [18]. Similarly, under flash viewing conditions where images are shown only briefly (an experimental manipulation that is close to the dynamic presentation of the CT images in this study), experts tend to miss subtle abnormalities more often than obvious ones [12]. It is likely that also in the current study, the more subtle abnormalities did not always receive foveal attention, which seems to be required for their detection, as they are hard to detect by parafoveal or peripheral vision.

One may ask then to what extent the experts in our study used the non-selective or global visual component in their search for abnormalities [2], [4]. In principle, more specific designs in combination with detailed analyses are needed to answer this question. However, what can be derived from the general distribution of fixations over the visual scene by experts is that they used the area anterior to the spine as an anchor point which they left when something suspicious presented itself in the parafovea or periphery, but to which they returned rapidly to inspect whether newly appearing lymph nodes were enlarged or not. We suspect that the non-selective visual component is actively in use when lymph nodes are not present or normal in size and least active (if it all) when lymph nodes in this area are indeed enlarged. The latter notion is supported by the successive small saccades and the increased number of fixations in the area anterior to the spine when ELNs were indeed present.

### Implications for the Interpretation of Eye-movement Measures

In several visual-search studies, standard eye-movement measures have been linked to the level of expertise. In their survey study, Gegenfürtner et al. [19] claimed that experts a) visit relevant areas more frequently than irrelevant areas; b) have on average shorter fixation durations; and c) make use of longer saccades. This eye-movement behaviour is, to their mind, the logical outcome of experts having larger retrieval structures and an extended perceptual span in their domain of expertise. In some domains, like reading, growing expertise is indeed consistently reflected in shorter fixation durations and longer saccadic amplitudes [23], [24], [38], [39]. The meta-analysis Gegenfürtner et al. [19] performed over a large numbers of studies indeed shows tendencies in these directions in the domain of visual search as well. Yet, if one considers individual studies, effects may go either way. For instance, in the domain of traffic, experts may revert to longer or shorter fixation durations than novices depending on the complexity of the traffic scene [25], [26]. Mann et al. [27] found that experts in sports typically use longer fixations than novices when performing or viewing a dynamic sports task/scene, but also that when viewing static stimuli, experts actually engage in shorter lasting fixations than novices. The current study adds to the evidence that in visual search, *one cannot directly link global measures to expert performance* (see [37], for a similar warning).

Thus, in contrast to the claims of Gegenfürtner et al. [19], we found that experts might visit relevant areas less frequently than non-experts. That is, in case of visually salient abnormalities, both semi-experts and naïve participants more frequently visited the relevant areas (the location where the abnormalities occurred) than experts. We also found that experts used fixation durations that were not shorter than those of non-experts (this especially holds for fixation durations in ELN videos; see Figure 5). Next we found that experts reverted to saccades of shorter amplitudes than non-experts (in all video types in this study, but especially in ABN videos). At the same time, we found that experts visited areas that were relevant for the ELN task more frequently than naïve participants and that those experts’ fixation durations were significantly shorter in ABN videos and normal videos than in ELN videos. In addition to this, we found that fluctuations in eye-movement behaviour even appeared in one and the same video as a consequence of the presence/absence of an abnormality. To summarize, our study adds to the evidence that it is counterproductive to look for a link between eye-movement measures and level of expertise on a global level. Instead, it suggest that one should profoundly contemplate the domain and context of the visual scene, the task that has to be performed, and the goals and strategies of the observers, before making predictions about eye movement behaviour as a function of expertise.

### Possible Implications for Radiologist Education

Previous work from our group has explored to what extent technical innovations (e.g., virtual microscopy) might benefit knowledge and skill development in medical education [40], [41]. The current study wanted to explore whether eye movement assessments during image perception may also be beneficial in medical education. The results of the current study suggest some possible applications.

In our study, experts outperformed the other groups in the ELN task and semi-experts outperformed naïve participants in the ABN task. This is in line with the notions that the more difficult a perceptual search task becomes, the more expertise is needed to deal with it successfully [19], [27]. This suggests that the manipulation of lesion subtlety and CT-image framerate will be a good tool to assess the stage of development of radiologists in training (residents). Good performance in tasks in which subtle lesions have to be detected (such as the ELN task) under high framerate conditions would indicate that residents have reached a high level of development. In line with the notion that increasing the framerate would affect the performance of people in an earlier stage of professional development, we found a minor, non-significant trend for the increasingly worse performance of semi-experts in the ELN task as the framerate increased (7 i/s: 61% correct; 14 i/s: 51% correct; 28 i/s: 48% correct) (Note that this is a trend that may turn out to be significant in future studies with more statistical power. In general the power of non-significant performance results was relatively low, between.08 and.15; for the example here it is actually.15. In other words, only quite sizable effects of performance could reach significance and possibly smaller effects, like the one here, may have been left undetected as a result of insufficient statistical power).

This study also showed that experts do not *necessarily* visit relevant areas more often than non-experts. That is, unlike the semi-experts and even naïve participants in our study, experts did not increase the number of fixations on areas where visceral abnormalities appeared. Given that their performance on these abnormalities was on a par with semi-experts and better than naïve participants, one should conclude that the experts needed relatively little visual input to reach the right assessment. Thus, instead that – as has been claimed repeatedly – more frequent visits to relevant areas is a landmark of a high level of expertise, exactly the opposite, namely, not increasing the frequency of visits to a relevant area, may be a sign of a high level of expertise. Thus the number of fixations on visually salient abnormalities can also be used as a measure of development in resident education: The fewer fixations needed to detect a visually salient abnormality, the more advanced the level of development.

Earlier studies also showed the possibilities of eye movements in skill development in the field of radiology. For instance, Litchfield and colleagues [42] showed that novices benefited from exposure to eye movements of radiographers or radiologists in the identification of pulmonary nodules during chest x-ray inspection. We believe that such studies alongside the current study may help to pave the way for the use of eye movements in the development of medical education.

### Conclusion

The main conclusion that can be drawn from this study is that expertise in radiology does not manifest itself in straightforward global patterns of eye movements. Yet experts’ eye-movement patterns are clearly different from those of semi-experts or from naïve viewers. In this case, we showed that – in response to the demands of two visually different tasks – experts differ from the other groups in their average fixation duration, saccadic amplitude and visits to relevant areas. More generally, it can be stated that expert behaviour is manifested in distinct eye-movement patterns of proactivity, reactivity and suppression, depending on the nature of the task, and the presence and type of abnormalities at any given moment.

## Supporting Information

Appendix S1 (DOCX)

## References

[1] Posner MI (1988) What is it to be an expert? In Chi MTH, Glaser R, Farr MJ, editors. The nature of expertise. Hillsdale, NJ: Lawrence Erlbaum. pp. xxix–xxxvi.

[2] Wolfe JM, Võ ML, Evans KK, Greene MR (2011) Visual search in scenes involves selective and nonselective pathways. Trends Cogn Sci 15: 77–84. 21227734 10.1016/j.tics.2010.12.001 PMC3035167

[3] Drew T, Evans KK, Võ ML, Jacobson FL, Wolfe JM (2013) What can you see in a single glance and how might this guide visual search in medical images? Radiographics 33: 263–274. 23104971 10.1148/rg.331125023 PMC3545617

[4] Nodine CF, Kundel HI (1987) The cognitive side of visual search in radiology. In O’Regan JK, Levy-Schoen A, editors. Eye movements: From physiology to cognition. Amsterdam: Elsevier. 573–582.

[5] Kundel HI, Nodine CF, Kunant EF, Weinhaus SP (2007) Holistic component of image perception in mammogram interpretation: Gaze-tracking study. Radiology 242: 396–402. 17255410 10.1148/radiol.2422051997

[6] Swensson RG (1980) A two-stage detection model applied to skilled visual search by radiologists. Percept Psychophys 27: 11–16.

[7] Miller G (1956) The magical number seven, plus or minus two: Some limits on our capacity for information processing. Psychol Rev 63: 81–97. 13310704

[8] Ericsson KA, Kintsch W (1995) Long-term working memory. Psychol Rev 102: 211–245. 7740089 10.1037/0033-295x.102.2.211

[9] Reingold EM, Sheridan H (2011) Eye movements and visual expertise in chess and medicine. In Liversedge SP, Gilchrist ID, Everling S, editors. Oxford handbook on eye movements. Oxford, UK: Oxford University Press. 767–786.

[10] Reingold EM, Charness N, Pomplun M, Stampe DM (2001) Visual span in expert chess players: Evidence from eye movements. Psychol Sci 12: 49–56. 10.1111/1467-9280.00309 11294228

[11] Kundel HI, Nodine CF (1975) Interpreting chest radiographs without visual search. Radiology 116: 527–532. 125436 10.1148/116.3.527

[12] Mugglestone MD, Gale AG, Cowley HC, Wilson ARM (1995) Diagnostic performance on briefly presented mammographic images. Proc SPIE 2436: 106–115.

[13] Carmody DP, Nodine CF, Kundel HI (1980) An analysis of perceptualand cognitive factors in radiographic interpretation. Perception 9: 339–344. 7454514 10.1068/p090339

[14] Krupinski EA, Tillack AA, Richter L, Henderson JT, Bhattacharyya AK, et al (2006) Eye-movement study and human performance using telepathology virtual slides: implications for medical education and differences with experience. Hum Pathol 37: 1543–1556. 17129792 10.1016/j.humpath.2006.08.024

[15] Manning D, Ethell S, Donovan T, Crawford T (2006) How do radiologists do it? The influence of experience and training on searching for chest nodules. Radiography 12: 134–142.

[16] Kocak E, Ober J, Berne N, Melvin WS (2005) Eye movement parameters correlate with level of experience in video-assisted surgery: Objective testing of three tasks. J Laparoendosc Adv Surg Tech A 15: 575–580. 16366861 10.1089/lap.2005.15.575

[17] Krupinski EA (1996) Visual scanning patterns of radiologists searching mammograms. Acad Radiol 3: 137–144. 8796654 10.1016/s1076-6332(05)80381-2

[18] Krupinski EA (2005) Visual Search of Mammographic Images: Influence of Lesion Subtlety. Acad Radiol 12: 965–969. 16023379 10.1016/j.acra.2005.03.071

[19] Gegenfurtner A, Lehtinen E, Säljö R (2011) Expertise Differences in the Comprehension of Visualizations: a Meta-Analysis of Eye-Tracking Research in Professional Domains. Educ Psychol Rev 23: 523–552.

[20] Ghirardelli ML, Jemos V, Gobbi PG (1999) Diagnostic approach to lymph node enlargement. Haematologica 84: 242–247. 10189390

[21] Jarodzka H, Scheiter K, Gerjets P, Van Gog T (2010) In the eyes of the beholder: How experts and novices interpret dynamic stimuli. Learn Instr 20: 146–154.

[22] Boshuizen HPA, Schmidt HG (1992) On the role of biomedical knowledge in clinical reasoning by experts, intermediates, and novices. Cogn Sci 16: 153–184.

[23] Rayner K (1998) Eye movements in reading and information processing: 20 years of research Psychol Bull. 124: 372–422. 10.1037/0033-2909.124.3.372 9849112

[24] Rayner K (2009) Eye movements and attention in reading, scene perception, and visual search. Q J Exp Psychol A 62: 1457–1506. 10.1080/17470210902816461 19449261

[25] Crundall D, Underwood G (1998) Effects of experience and processing demands on visual information acquisition in drivers. Ergonomics 41: 448–458.

[26] Underwood G, Chapman P, Brocklehurst N, Underwood J, Crundall D (2003) Visual attention while driving: Sequences of eye fixations made by experienced and novice drivers. Ergonomics 46: 629–646. 12745692 10.1080/0014013031000090116

[27] Mann DTY, Williams AM, Ward P, Janelle CM (2007) Perceptual-cognitive expertise in sport: A meta-analysis. J Sport Exerc Psychol 29: 457–478. 17968048 10.1123/jsep.29.4.457

[28] Miura T (1990) Active function of eye movement and useful field of view in a realistic setting. In Gröner R, d’Ydewalle G, Parnham R, editors. From eye to mind: Information acquisition in perception, search and reading. Amsterdam: Elsevier. 119–127.

[29] Law B, Atkins MS, Kirkpatrick AE, Lomax AJ, Mackenzie CL (2004) Eye gaze patterns differentiate novice and experts in a virtual laparoscopic surgery training environment. In Duchowski A, Vertegan R, editors. Proceedings of the 2004 symposium on eye tracking research and applications. San Antonio, TX: Association for computing machinery. 41–48.

[30] Wilson M, McGrath J, Vine S, Brewer J, Defriend D, et al (2010) Psychomotor control in a virtual laparoscopic surgery training environment: gaze control parameters differentiate novices from experts. Surg Endosc 24: 2458–2464. 20333405 10.1007/s00464-010-0986-1 PMC2945464

[31] Balslev T, Jarodzka H, Holmqvist K, De Grave W, Muijtjens AMM, et al (2011) Visual expertise in paediatric neurology. Eur J Paediatr Neurol 16: 161–166. 21862371 10.1016/j.ejpn.2011.07.004

[32] Godwin HJ, Menneer T, Cave KR, Donnelly N (2010) Dual-target search for high and low prevalence X-ray threat targets. *Visual Cognition*, *18*, 1439–1463.

[33] Baayen RH (2008) Analyzing linguistic data: A practical introduction to statistics. Cambridge: Cambridge University Press. 368 p.

[34] Pinheiro JC, Bates DM (2000) Mixed-effects models in S and S-PLUS: Statistics and computing. New York: Springer. 528 p.

[35] R Development Core Team (2007) R: A language and environment for statistical computing. Vienna, Austria: Available: http://www.R-project.org.

[36] Baayen RH, Davidson DJ, Bates DM (2008) Mixed effects modeling with crossed random effects for subjects and items. J Mem Lang 59: 390–412.

[37] Donovan T, Litchfield D (2012) Looking for cancer: Expertise related to differences in searching and decision making. App Cogn Psychol: doi:10.1002/acp.2869

[38] Häikiö T, Bertram R, Hyönä J, Niemi P (2009) Development of the letter identity span in reading: Evidence from the eye movement moving window paradigm. J Exp Child Psychol 102: 167–181. 18538339 10.1016/j.jecp.2008.04.002

[39] Häikiö T, Bertram R, Hyönä J (2011) The development of whole-word representations in compound word processing: Evidence from eye fixation patterns of elementary school children. Appl Psycholinguist 32: 533–551.

[40] Helle L, Nivala M, Kronqvist P, Gegenfurtner A, Björk P, et al.. (2011) Traditional microscopy instruction versus process-oriented virtual microscopy instruction: A naturalistic experiment with control group. Diagn Pathol: doi:10.1186/1746-1596-6-S1-S8 10.1186/1746-1596-6-S1-S8 PMC3073226 21489203

[41] Helle L, Säljö R (2012) Collaborating with digital tools and peers in medical education: cases and simulations as interventions in learning. Instr Sci 40: 737–744.

[42] Litchfield D, Ball LJ, Donovan T, Manning DJ, Crawford T (2010) Viewing another person’s eye movements improves identification of pulmonary nodules in chest x-ray inspection. J Exp Psychol Appl 16, 251–262. 10.1037/a0020082 20853985

