# Supplementary material for: The Effect of Expertise on Eye Movement Behaviour in Medical Image Perception
Source: PLoS One. 2013 Jun 13;8(6):e66169. doi: 10.1371/journal.pone.0066169 (PMC3681771; doi:10.1371/journal.pone.0066169)
Supplement: Appendix S1 — (DOCX) [file pone.0066169.s001.docx]

APPENDIX 1

In the tables we present the fixed effects of the final models for the experiment with only those effects or interactions reported that significantly (p < 0.05) improved the model’s performance as indicated by the likelihood ratio model comparison tests. If an interaction was found significant, its main effects are reported as well: we note that these effects are not independently interpretable in the lmer() output.

Key to tables: MCMC = Monte Carlo Markov chain; HPD95lower = lower boundary of the 95% highest posterior density interval; HPD95upper = upper boundary of the 95% highest posterior density interval; pMCMC = p values estimated by the MCMC method using 10,000 simulations; pr(>|t|) = p-values obtained with t-test using the difference between the number of observations and the number of fixed effects as the upper bound for the degrees of freedom

Table A. Fixed effects of all significant factors and interactions for average fixation durations across videos

| Fixed effects | Estimate | MCMCmean | HPD95lower | HPD95upper | pMCMC | Pr(>\|t\|) |
| --- | --- | --- | --- | --- | --- | --- |
| (Intercept) | 2.4992 | 2.4994 | 2.4420 | 2.5564 | 0.0001 | 0.0000 |
| ExpertSE | 0.0357 | 0.0352 | -0.0353 | 0.1049 | 0.3240 | 0.3264 |
| ExpertN | 0.0208 | 0.0206 | -0.0384 | 0.0834 | 0.4856 | 0.5073 |
| VideoABN | -0.0112 | -0.0113 | -0.0514 | 0.0282 | 0.4318 | 0.2881 |
| VideoELN | 0.0262 | 0.0262 | -0.0115 | 0.0676 | 0.1196 | 0.0158 |
| FRate14 | -0.0007 | -0.0007 | -0.0061 | 0.0043 | 0.7834 | 0.7957 |
| FRate28 | 0.0154 | 0.0155 | 0.0089 | 0.0220 | 0.0001 | 0.0000 |
| ExpertSE:VideoABN | -0.0057 | -0.0057 | -0.0210 | 0.0108 | 0.4796 | 0.4832 |
| ExpertN:VideoABN | 0.0068 | 0.0069 | -0.0069 | 0.0202 | 0.3234 | 0.3192 |
| ExpertSE:VideoELN | -0.0184 | -0.0184 | -0.0354 | -0.0020 | 0.0314 | 0.0328 |
| ExpertN:VideoELN | -0.0215 | -0.0215 | -0.0358 | -0.0064 | 0.0024 | 0.0036 |

*Note.* In the model presented here we used ExpertE, VideoNorm and FRate7 as a baseline.

##### Table B. Fixed effects of all significant factors and interactions for average saccadic amplitude across videos

| Fixed effects | Estimate | MCMCmean | HPD95lower | HPD95upper | pMCMC | Pr(>\|t\|) |
| --- | --- | --- | --- | --- | --- | --- |
| (Intercept) | 1.3295 | 1.3296 | 1.2544 | 1.4122 | 0.0001 | 0.0000 |
| ExpertE | -0.0851 | -0.0851 | -0.1570 | -0.0131 | 0.0238 | 0.0223 |
| ExpertN | 0.0414 | 0.0414 | -0.0158 | 0.0978 | 0.1500 | 0.1568 |
| VideoNorm | -0.0735 | -0.0737 | -0.1613 | 0.0132 | 0.0788 | 0.0032 |
| VideoELN | -0.0898 | -0.0900 | -0.1857 | 0.0045 | 0.0546 | 0.0010 |
| FRate14 | -0.0134 | -0.0136 | -0.0318 | 0.0039 | 0.1406 | 0.1420 |
| FRate28 | 0.0067 | 0.0064 | -0.0132 | 0.0262 | 0.5062 | 0.4994 |
| ExpertE:VideoNorm | 0.0499 | 0.0500 | 0.0263 | 0.0742 | 0.0001 | 0.0000 |
| ExpertN:VideoNorm | 0.0198 | 0.0199 | 0.0012 | 0.0391 | 0.0384 | 0.0419 |
| ExpertE:VideoELN | 0.0316 | 0.0318 | 0.0038 | 0.0580 | 0.0236 | 0.0232 |
| ExpertN:VideoELN | 0.0121 | 0.0122 | -0.0090 | 0.0330 | 0.2586 | 0.2651 |
| ExpertE:FRate14 | 0.0248 | 0.0249 | -0.0006 | 0.0505 | 0.0608 | 0.0589 |
| ExpertN:FRate14 | 0.0284 | 0.0286 | 0.0089 | 0.0505 | 0.0080 | 0.0075 |
| ExpertE:FRate28 | 0.0204 | 0.0205 | -0.0095 | 0.0497 | 0.1732 | 0.1753 |
| ExpertN:FRate28 | -0.0003 | -0.0002 | -0.0240 | 0.0227 | 0.9902 | 0.9802 |

##### Note. In the model presented here we used ExpertSE, VideoABN and FRate7 as a baseline.

Table C. Fixed effects of all significant factors and interactions for average saccadic amplitude in ELN-videos

| Fixed effects | Estimate | MCMCmean | HPD95lower | HPD95upper | pMCMC | Pr(>\|t\|) |
| --- | --- | --- | --- | --- | --- | --- |
| (Intercept) | 1.0431 | 1.0489 | -0.0299 | 1.9805 | 0.0458 | 0.0000 |
| ExpertSE | 0.1733 | 0.1724 | 0.0808 | 0.2585 | 0.0001 | 0.0001 |
| ExpertN | 0.2125 | 0.2115 | 0.1344 | 0.2884 | 0.0001 | 0.0000 |
| ELN_No | 0.1332 | 0.1332 | 0.0880 | 0.1734 | 0.0001 | 0.0000 |
| FRate14 | 0.0229 | 0.0206 | -0.0293 | 0.0659 | 0.3896 | 0.3514 |
| FRate28 | 0.0654 | 0.0631 | 0.0213 | 0.1056 | 0.0044 | 0.0024 |
| ExpertSE:ELN_No | -0.1253 | -0.1253 | -0.1818 | -0.0716 | 0.0002 | 0.0000 |
| ExpertN:ELN_No | -0.1121 | -0.1120 | -0.1606 | -0.0643 | 0.0001 | 0.0000 |
| ExpertSE:FRate14 | -0.0245 | -0.0245 | -0.0920 | 0.0391 | 0.4638 | 0.4611 |
| ExpertN:FRate14 | -0.0101 | -0.0095 | -0.0617 | 0.0455 | 0.7258 | 0.7132 |
| ExpertSE:FRate28 | -0.0647 | -0.0631 | -0.1180 | -0.0067 | 0.0274 | 0.0219 |
| ExpertN:FRate28 | -0.0677 | -0.0670 | -0.1147 | -0.0175 | 0.0076 | 0.0069 |

##### * In the model presented here we used ExpertE, ELN_Yes and FRate7 as a baseline.

##### Table D. Fixed effects of all significant factors and interactions for average saccadic amplitude in ABN-videos

| Fixed effects: | Estimate | MCMCmean | HPD95lower | HPD95upper | pMCMC | Pr(>\|t\|) |
| --- | --- | --- | --- | --- | --- | --- |
| (Intercept) | 1.3164 | 1.3069 | -0.0638 | 2.9295 | 0.0662 | 0.0000 |
| ExpertSE | 0.0884 | 0.0887 | -0.0182 | 0.2015 | 0.1086 | 0.1150 |
| ExpertN | 0.1492 | 0.1501 | 0.0563 | 0.2405 | 0.0020 | 0.0014 |
| ABN_No | 0.0031 | 0.0031 | -0.0303 | 0.0354 | 0.8584 | 0.8562 |
| FRate7 | -0.0520 | -0.0504 | -0.0948 | -0.0022 | 0.0304 | 0.0268 |
| FRate28 | -0.0444 | -0.0434 | -0.0977 | 0.0121 | 0.1200 | 0.1128 |
| ExpertSE:ABN_No | -0.0695 | -0.0695 | -0.1136 | -0.0234 | 0.0020 | 0.0025 |
| ExpertN:ABN_No | -0.0547 | -0.0548 | -0.0924 | -0.0162 | 0.0042 | 0.0051 |
| ExpertSE:FRate7 | 0.0640 | 0.0640 | -0.0006 | 0.1299 | 0.0576 | 0.0541 |
| ExpertN:FRate7 | 0.0398 | 0.0392 | -0.0106 | 0.0895 | 0.1308 | 0.1183 |
| ExpertSE:FRate28 | 0.0758 | 0.0746 | 0.0015 | 0.1514 | 0.0480 | 0.0497 |
| ExpertN:FRate28 | 0.0740 | 0.0735 | 0.0106 | 0.1370 | 0.0242 | 0.0235 |

* In the model presented here we used ExpertE, ABN_Yes and FRate14 as a baseline.
